# Providing information about medication changes upon discharge from a geriatric unit: the community healthcare professionals’ point of view

**DOI:** 10.1186/s12877-023-04551-4

**Published:** 2024-01-09

**Authors:** Céline Rahuel, Maxime Pautrat, Amal Aïdoud, Bertrand Fougère, Camille Debacq

**Affiliations:** 1 Division of Geriatric Medicine, Tours University Hospital, 37000 Tours, France; 2 Department of General Practice, Tours University Hospital, 37000 Tours, France; 3 EA7505 (Education, Ethics, Health), University of Tours, Tours, France; 4 EA4245 (Transplantation, Immunology, Inflammation), University of Tours, Tours, France

**Keywords:** Adverse drug event, Medication reconciliation, Community-hospital communication, Geriatrics, Geriatric medicine

## Abstract

**Introduction:**

It is well known that polypharmacy is associated with adverse drug events. Accordingly, specialist geriatric units have to pay particular attention to the appropriateness of prescription and the withdrawal of potentially inappropriate medications. Even though community healthcare professionals are keen to received medication reconciliation results, the literature data show that the quality of communication between the hospital and the community needs to be improved.

**Objective:**

To assess community healthcare professionals’ opinions about the receipt of medication reconciliation results when a patient is discharged from a specialist geriatric unit.

**Method:**

We performed a qualitative study of general practitioners, community pharmacists and retirement home physicians recruited by phone in the Indre-et-Loire region of France. A grounded theory method was used to analyze interviews in multidisciplinary focus groups.

**Results:**

The 17 community healthcare professionals first explained why the receipt of medication reconciliation results was important to them: clarifying the course and outcomes of hospital stays and reducing the lack of dialogue with the hospital, so that the interviewees could provide the care expected of them. The interviewees also described mistrust of the hospital and uncertainty when the modifications were received; these two concepts accentuated each other over time. Lastly, they shared their opinions about the information provided by the hospital, which could improve patient safety and provide leverage for treatment changes but also constituted a burden.

**Perspectives:**

Our participants provided novel feedback and insight, constituting the groundwork for an improved medication reconciliation form that could be evaluated in future research. Exploring hospital-based professionals’ points of view might help to determine whether the requested changes in the medication reconciliation form are feasible and might provide a better understanding of community-to-hospital communication.

## Introduction

The prevalence of adverse drug events (ADEs) increases with the patient’s age [1]; this is notably the case after the age of 65, due (in part) to slower metabolism and a greater prevalence of polypharmacy (defined as taking five or more medications during the same period) [2, 3]. ADEs are responsible for 5 to 10% of hospital admissions among patients aged 65 and over and for 20% among patients in their eighties [4]. Furthermore, polypharmacy is observed in up to 15% of patients attending the emergency department [5]. Lastly, polypharmacy is involved in 2 to 12% of in-hospital deaths and increases the risk of falls by 21% [6]. Importantly, 30 to 60% of ADEs are preventable [7].

ADEs can be prevented by reducing the prescription of potentially inappropriate medications (PIMs); it has been estimated that reducing the number of PIMs by 10% leads to a 60% decrease in the number of ADEs [8]. In this respect, specialist geriatric units must pay particular attention to good prescribing practices and the reduction in PIMs. Various tools for improving medication appropriateness have been developed, such as the Laroche list and the STOPP-START criteria [9, 10]. These tools have proved to be effective: when used in specialist geriatric units, they lead to a treatment change for 80% of patients and are associated with a lower number of PIMs, fewer ADEs, and a shorter length of hospital stay [11, 12].

Hence, deprescribing via application of the STOPP-START criteria can lead to many treatment changes. These changes are passed on to the patient’s general practitioner (GP) and community pharmacist via medication reconciliation.

Medication reconciliation involves a comparison of the patient’s treatments on admission with those prescribed upon discharge. This comparison is sent at discharge to transmit medication changes. The patient’s treatments on admission comprise his/her prescription medications and any over-the-counter medications used for self-medication. The level of treatment compliance is also noted. Information about the patient’s medications must be gathered from at least two different sources [13].

Medication reconciliation is usually well received by community healthcare professionals; the information is relevant and useful, and so most medication reconciliations are implemented after the patient has been discharged [14, 15].

However, many studies have shown that communication between hospital and the community healthcare professionals is not optimal and could be improved by focusing on the transmission of useful, adequate information [16, 17]. Hospital-community collaboration is seen as being good for the patient, and GPs want to be involved in the medication reconciliation process [18, 19].

However, few studies have questioned community healthcare professionals (and especially community pharmacists) about their expectations, and the few data available in the literature were collected via surveys.

By involving all the healthcare professionals involved in medication reconciliation, our long-term goal is to develop better tools and improve the implementation of treatment changes. The objective of the present study was to explore community healthcare professionals’ opinions on the community transmission of medication changes when a patient is discharged from a specialist geriatric unit.

## Methods

### Study design

The study was conducted by two females a GP intern and a hospital geriatrician. We organized multidisciplinary focus groups by using a qualitative, inductive method to gain fresh perspectives and collect new ideas and innovative concepts. Dynamic focus groups provide rich discussions and ensure that several points of view can be expressed [20, 21]. We then developed a theory by conceptualizing the points of view expressed in the focus group [22].

### Participants

We included the community healthcare professionals most frequently involved in medication reconciliation (i.e. general practitioners and pharmacists), together with people who occasionally worked with medication reconciliation (e.g. locum GPs and pharmacists, and physicians working in retirement homes). Firstly, we contacted professionals who had received medication reconciliation results from Tours University Hospital (Tours, France) in the previous three months. The participant were not expert in MR ensuring from the field opinion. To ensure sample diversity, participants were recruited by phone, via e-mail and by snowball sampling.

### Data collection

The group interviews were semistructured, with the use of a guide with open questions comprising three themes: experience, difficulties, and suggestions with MR. The guide was not provided to the participants. Every interview started with the question “describe how you prescribe new medication or refill a new prescription on a daily basis, for an elderly patient”. Given that our study took place during the COVID-19 pandemic, the interviews were conducted with Zoom® video conferencing software (Zoom Video Communications, Inc., San Jose, CA, USA). The interview guide was drawn up by the investigators and could be amended according to how the interviews went. One investigator led the interviews, while a second investigator noted non-verbal communication. The presentation of medication reconciliations issued by other hospitals could also be used to stimulate discussion.

### Analysis

The interviews were transcribed manually by the investigators. The first two interviews were annotated independently by two investigators, who then resolved any differences by consensus. We used a procedure based on a grounded theory analysis to conceptualize the participants’ points of view [22]. All the interview excerpts presented below were translated from French by a native English speaker, in order to preserve the meaning.

### Ethical issues

In line with the French legislation on noninterventional studies of clinical practice, the study protocol was approved by the Ethics Committee in Human Research of the University Hospital of Tours, France. Oral informed consent, approved by the Ethics Committee in Human Research of the University Hospital of Tours, France, was also obtained at the beginning of each interview.

## Results

### The focus groups

We organized five focus groups, so that we had enough data to build an explanatory model. We reached data saturation after four interviews and led a fifth to ensure data saturation. Each focus group had three to four participants, giving a total of 17 (two physicians from retirement homes, seven pharmacists, and eight GPs). Thirteen of the 17 participants (76.6%) were under 39 years of age, and nine of the participants (52.9%) were women. The shortest interview lasted 30 min and the longest one hour.

### Analysis: major themes

#### A relationship rooted in mistrust

Firstly, the participants described their mistrust of the hospital, the decisions made, and the work actually performed.

This mistrust was rooted in previous bad experiences: *“Sometimes on discharge, the patient ends up with two anticoagulants, sometimes none at all, and sometimes we only see enoxaparin (I mean an injectable) and no follow-on treatment”* (focus group (FG) 4, community pharmacist (CP) 2).

The mistrust was sometimes rooted in the interviewees’ representations of the work performed in the hospital: *“It’s true that sometimes we have medications that have been stopped for discharged patients and we wonder whether it was a mistake: a medical student did the discharge and typed up the discharge note as quickly as possible so that the patient could get out or, uh, simply that the senior physician didn’t notice that there had been a change and that a treatment had been forgotten”* (FG1, GP1).

The interviewees also thought that they knew the patient better than hospital-based professionals did. This feeling made them doubt whether the hospital had all the information needed to make the best decisions: *“Unfortunately, when they are hospitalized, you might not have all the information, so when a treatment is missing we don’t know whether that’s because a prescription from a specialist (in addition to the prescription from the GP) is missing or if it was intentional”* (FG2, CP2).

Given that the interviewees did not always see the reconciliation after a patient’s discharge, they sometimes wondered why it was sent: *“It might get someone off the hook; they could say “Well, I sent it, it’s done””* (FG5, GP1).

#### Community healthcare professionals are heard but not listened to

Our participants described a lack of dialogue between the hospital and the community. They had trouble contacting people at the hospital, either because they could not identify the right person* (“We always have trouble contacting the hospital because the name of the house officer who wrote the prescription is not always given – sometimes it’s just written “house officer”, so we don’t know who to contact”* (FG4, CP2)) or because of organizational problems (*“On a daily basis, it takes a huge amount of time to find the right person because we’re not calling at the right time of day, so we’re going to end up with the secretary who doesn’t have the information, who’s just going to pass the buck on to someone else, but the house officer isn’t there. Or it was a medical student but they’ve left since…” (*FG4, CP2)). Lastly, the mistrust can be due to the participant him/herself: *“It’s not always easy to call and bother a hospital physician to find out which changes were really made or not”* (FG4, CP2).

In contrast to the pharmacists, the GPs in the focus groups wanted to give their opinion on treatment changes; the lack of dialogue with hospital staff made that difficult: *“This way we can call the hospital back; as a physician, I want to know why there were changes and I want to decide for myself (when I see the patient) whether the reason is valid and whether I actually want to continue to prescribe the medication”* (FG3, GP1).

#### When medication reconciliation perturbs the community recipient

The community healthcare professionals were sometimes perturbed by receipt of the medication reconciliation results. On one hand, this perturbation could have a technical cause: the tool was difficult to incorporate into their professional software and work routine: *“From the pharmacist’s perspective, and I’m speaking for my colleagues, it’s not something we know how to integrate into our software. We can’t really use it to fill prescriptions or… The best we can do with this tool is put a memo on the patient’s records that says “Be careful: treatment change” but that may or may not be seen by the pharmacy technician or the pharmacist who fills the next prescription”* (FG5, CP2).

On the other hand, the perturbation could also be psychological and could lead to a chain reaction. Receipt of the reconciliation results could create doubt in the GPs’ minds: *“sometimes when a discharged patient’s treatment is withdrawn, we wonder whether it’s a mistake or not”* (FG1, GP1) and for pharmacists *“It’s true that for the important medications, we always wonder whether the change was intentional or not”* (FG4, CP1). This uncertainty and the perceived lack information can prevent the healthcare professional from doing his/her job *“The infamous Friday discharge when we don’t get the information until, say, Saturday afternoon… and if something needs to be done on Saturday afternoon, we can’t do it”* (FG3, CP2).

All of the above factors caused uneasiness because the healthcare professionals felt that they could not do their job properly or answer the patient’s questions. This feeling was shared by GPs and pharmacists: *“We still see differences between discharge prescriptions where it’s written that medications are withdrawn and replaced and prescriptions where we just have a list of medications, and when one has been changed or is missing, we don’t know if it’s intentional or not. That kind of thing can often be uncomfortable”* (FG4, CP2). This uneasiness prompted the community healthcare professionals to look for answers at the hospital: *“When we only have a prescription with information that can seem incomplete, it might be either intentional, an oversight or a modification, and we have to call the hospital to ask. But it’s not always easy to get an answer”* (FG5, GP1). The community healthcare professionals also asked the patients: *“So, when we have questions about the prescription, we can ask them [the patients] but they don’t know either”* (FG4, CP2).

#### Information for greater safety

Information can reassure the recipient: *“I’ve seen this type of thing [the medication reconciliation results] before. For patients with polypharmacy, it was useful to be sure that nothing had been forgotten”* (FG1, GP1). Furthermore, information can make him/her more vigilant: *“It can be useful to know the results of the kidney function tests when analyzing the medications, uh, it’s not calculated often but, uh, perhaps we should put a warning (in the patient’s file) or something”* (FG4, CP2).

The GPs and pharmacists considered that the provision of information can ensure a better continuum of care: *“It is really useful to know why our patients have been admitted and where they’ve been discharged, that they’ve been hospitalized or have gone home. We often get it [the medication reconciliation form] before we see the patient, so we can prepare the next consultation”* (FG4, GP1). *“To do a good job, we should get it [the medication reconciliation form] the day before [hospital discharge]”*(FG4, GP2).

#### Information as leverage for implementing treatment changes

The community healthcare professionals could use the medication reconciliation results as leverage for treatment changes. For example, it could ensure that the patients applied the medication changes in the long term: *“There are a fair amount of patients who, upon discharge, have a lot less prescriptions, and we often have pressure put on us to go back to the previous treatment, and having very clear explanations and comments about the modifications, and being sure that they were intended, makes it a very good tool”* (FG5, GP1), *“I'm not saying that there is a need to establish it, but it allowed me to re-explain what had been decided and therefore to support it”* (FG3, CP2).

The medication reconciliation results can also help to initiate a discussion between professionals about the patient: *“I really like this kind of document: it makes discussions with GPs easier. When an atropine-like medication or some other prescription drug that appears to be inappropriate is withdrawn, the document allows us to point that out to the GP”* (FG2, retirement home physician 2).

#### Patient empowerment

Some healthcare professionals said that seeing the medication reconciliation results might make a patient less passive: *“It might be a good thing* for *patients to take the form home with them, for example. Especially if it’s well done, it could give them a better understanding [of their treatments] too*” (FG1, GP1).

#### Information as a burden

An unappealing tool could be seen as a burden: *“too much information kills the information”* (FG3, CP2)*, “A lot of information about medications (in the sense that it's good to state, for example, that the original brand-name drug in Pevaryl® 1% cream is econazole), that's good, but all the things we already know and that are of no interest make the process more cumbersome”* (FG3, CP3).

#### The need for adequate information

The information has to be adequate so that it is not considered as a burden: *“I think the key information is which medications are kept and which ones we’re going to have to renew and represcribe afterwards*” (FG4 GP3). *“We often don’t have the exact dates or the reasons on the anticoagulants [to be prescribed after discharge], it’s often complicated”* (FG4, CP1).

#### The right moment…

Our participants mentioned that the time when they receive the medication reconciliation results is crucial. The results are only useful when they are timely: *“The benefit of this is that if it’s well done (and if I have understood correctly, it’s immediate), it explains the thought processes and can be very useful for the pharmacist, the patient, and the GP—if it arrives quickly enough”* (FG2, retirement home doctor 2).

#### …differs from one profession to another

The professionals’ views differed with regard to the right moment for receiving the medication reconciliation results. One participant summarized this divergence as follows: *“Earlier, we talked about the moment when the reconciliation is sent. According to Dr 1 (GP), it was satisfactory because he received the results in a few days, before the patient was discharged with their medications, *etc*. And so he had time to receive the results and see the patient. Whereas because we’re dealing with the discharge prescription, we need to have the reconciliation results immediately, and if there’s a modification, we can do it straight away. So I think the different points of view are linked to our respective roles as GPs and pharmacists”* (FG3, CP2).

#### A wish for modern, speedy transmission

The participants mentioned e-mail as the most appropriate way of sending the medication reconciliation forms – notably with regard to speed and traceability: *“In fact, I really think that IT is a quite simple, rapid fast way for both sides. The writer just has to click on “save” or “send” somewhere and we receive it automatically”* (FG4 GP1). *“I really prefer secure messaging for its traceability and the ability to search and everything—it’s much more practical for me”* (FG3, CP2).

#### Less is more

The interviewed healthcare professionals wanted a medication reconciliation form that could be understood in a glance: *“[the form’s format] seems clear to me. It summarizes things, that’s what’s good. In a glance, we can see the before, the after, and why. It’s good”* (FG1, GP5).

In particular, they mentioned a non-dense presentation: *“I think a medication reconciliation form needs to be to the point so that it gives the information in the simplest, clearest way possible”* (FG4, CP3).

A universally understood color code appears to be essential: *“The use of color, for example. If red means it has been withdrawn, then it’s immediately clear. And if green is continuation, it’s obvious. We’re used to seeing these color codes for things we have to pay attention to: green means it was appropriate and still is”* (FG3, GP1).

## Discussion

### Summary of the results

The study participants described their mistrust of the hospital and how they were perturbed by the receipt of medication reconciliation results. However, they explained that transmission of medication changes is important for clarifying the course and outcomes of hospital stays and countering the lack of hospital-community dialogue. Medication reconciliation provided the participants with tools to do their job. The participants viewed the transmitted information as promoting safety, leverage for treatment changes, and empowerment but also as a burden.

### Comparison of the major themes with literature

#### Dynamic interactions between mistrust and perturbation

The primary reason for doubt and concern among community healthcare professionals was technical: the format of the medication reconciliation results was difficult to incorporate into the professionals’ software and their work routine.

**Fig. 1:**
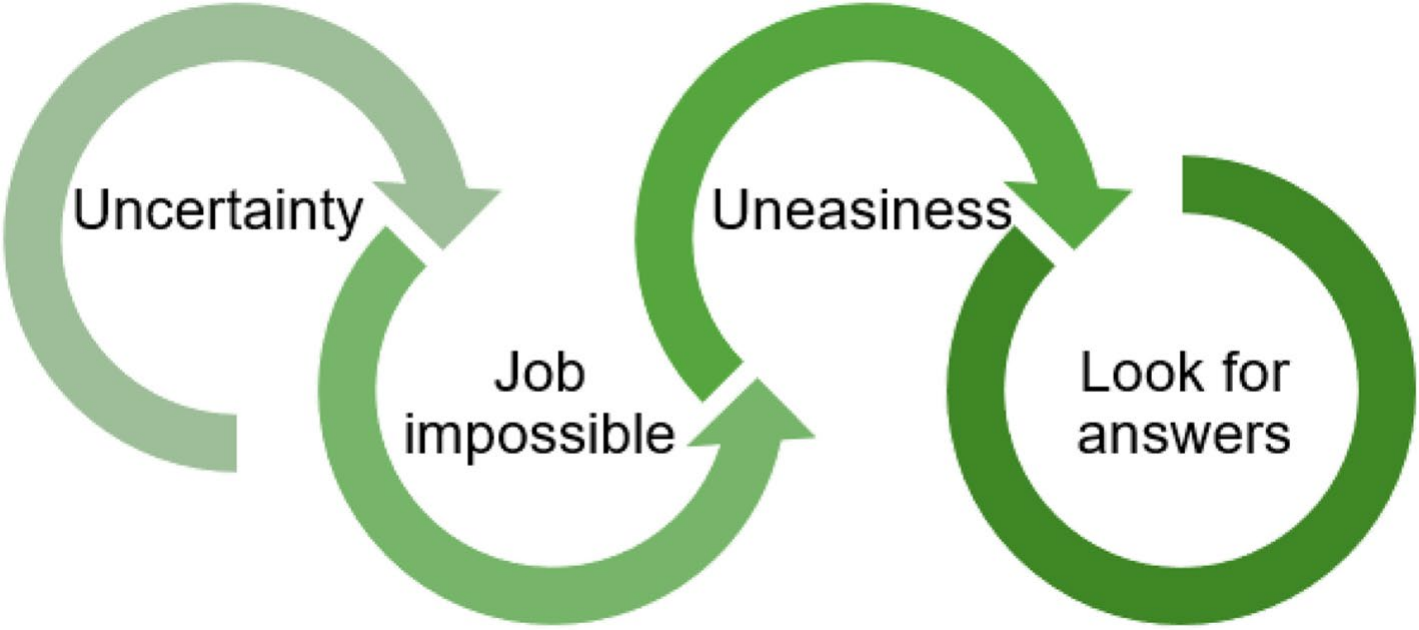
Psychological perturbations

The transmitted information could sometimes appear to be incomplete or wrong, which created uncertainty. Community healthcare professionals also felt that they knew the patient better than the hospital staff did. This feeling created doubts about decisions made in the hospital: “Did they have all the information?”. This uncertainty created uneasiness in the healthcare professional’s practice and prevented them from doing their work as they wanted to (filling the prescription, preparing the next consultation, and answering the patient's questions). These factors prompted the healthcare professional to search for answers, and the psychological disturbance led to a chain reaction (Fig. 1).

#### A lack of dialogue

Obtaining answers about medication reconciliation was difficult, due to the perceived imbalance in community-hospital dialogue. Community healthcare professionals had difficulty contacting hospital-based professionals, and hospital-based professionals often did not reach out to community healthcare professionals.

In the present study, community healthcare professionals stated that they were not informed systematically when one of their patients was hospitalized or when the patient was discharged or died in hospital. In her thesis in 2015 “*Enquête auprès des médecins généralistes sur l’apport des nouvelles technologies dans la relation médecine de ville-hôpital*”*,* Delahaye reported that 51% of the doctors questioned were not informed of their patient's hospitalization, and 45% were only informed during the stay. As for discharge, 55% were informed after discharge (by the hospitalization report) and 33% were not informed at all. Comparable results were found in other studies in 2006 and 2014 [23, 24].

Literature also shows they is no improvement over the last decade: for an event such as death for example, 12% of GPs were informed in 2006 and 17% in 2018 [23, 25]. In most cases, GPs are told about a patient’s death by the latter’s family (rather than the hospital), which creates poor experiences with regard to the hospital discharge process.

When a hospital contacts a GP, it is usually to obtain additional information about a hospitalized patient (78.9% of instances, according to Lacharme’s report in 2018) [25]. The same applies to pharmacists, who report that they are usually contacted for more information about the patient’s medication. This reinforces the impression of a lack of dialogue after the reconciliation results have been received.

The one-sidedness and difficulties of community-hospital communication has been widely described in the literature. As early as 1993, sociologists described the “absence of reciprocity” felt by GPs interviewed about communication with the hospital [26].

Following repeated requests for easier communication, Lille University Hospital (Lille, France) set up the Hop'Line system in 2008 [27]. This toll-free phone line (available from 9am to 7 pm, Monday to Friday) enables GPs to contact a specialist at the hospital directly. The GPs’ feelings about this initiative have been reported in A. Lemoine thesis in 2017 “*Collaboration interprofessionnelle entre médecins généralistes et praticiens hospitaliers du CHRU de Lille. Exemple de la Hop’Line*”*.* It is noteworthy that despite the efforts made by the hospital, the complaints made (e.g. difficulty contacting people, and not knowing who to contact) were the same as before the hotline opened.

These elements indicate that community-hospital communication is a question of wanting to communicate and not just being able to do so. This raises the question of whether hospital-based healthcare professionals want to provide documents and information. However, to the best of our knowledge, hospital-based physicians have rarely or never been questioned about community-hospital communication.

These concerns and their consequences affected the community-hospital relationship by creating poor post-discharge experiences. The vicious circle between destabilization and mistrust must be taken into account when seeking to understand the processes at work when medication reconciliation results are received*.*

In that regard in 2018, Social Security financing law’s article 51 offered new financing for innovative health projects about integrative care pathways [28]. These projects are about highly diverse health issues such as COVID-19, pediatric obesity or depression [29]. This demonstrates the issues described earlier are universal and not specific to MR. The link between these projects is a common will to change practice and better communication, in order to benefit the patients.

#### Knowledge is power

Most of the community healthcare professionals interviewed in the present study had a positive view of the post-discharge transmission of medication reconciliation results. The healthcare professionals mention the safety that the transmission provided, which led to greater vigilance and reassurance and enabled them to provide uninterrupted care. The interviewees also saw this transmission as a lever for reinforcing the treatment changes initiated during the hospital stay. In this respect, smooth transmission of the reconciliation results to community healthcare professionals probably helps to reduce the number of PIMs and ADEs in the long term.

The two main complaints made by the GPs interviewed here were a lack of information and information not being provided on time. The timely provision of medication reconciliation results would therefore empower the recipients, help them to do their job the way they want to, sustain treatment changes, and reduce the incidence of ADEs.

### Perspectives

The study participants provided novel feedback on what they want and need from the medication reconciliation process. Our findings might help to improve medication reconciliation tools, which could then be evaluated in future research.

By questioning the study participants about a clinical tool (a paper medication reconciliation form), we were able to explore their relationship with the hospital. The difficulties of community-hospital communication have been widely described in the literature. The community healthcare professionals interviewed here considered that two-way communication (i.e. dialogue with hospital staff) is essential for their practice but is currently lacking. It would be interesting to (i) explore the views of hospital-based healthcare professionals on community-hospital communication and (ii) determine whether the changes requested by the community healthcare professionals surveyed in the present study are feasible.

### Limitations and strengths

The study population was young, and there were no participants in the 50–59 age group. According to a 2021 study by the French Ministry of Health’s statistics office (*Direction de la Recherche, des Etudes, de l’Evaluation et des Statistiques*), the mean age for practicing pharmacists is 46.3, and that of GPs is 49.3 [30].

Furthermore, two of the interviewed physicians worked in a retirement home. The comments during the first focus group made us realize that these physicians did not prescribe medications and so did not deal with the post-discharge treatment changes. We therefore decided to stop recruiting this type of physician.

Firstly, the use of video conferencing might have dampened the group dynamics, and so some participants might have held back from saying everything they wanted to. Secondly, the presence of an observer (a physician from the university hospital) might also have prevented some participants from speaking out about a tool developed by the university hospital. To minimize this bias, the physician leading the focus group turned off her microphone and her camera during the interviews, which gave the participants the impression that she was not present. Thirdly, and as with all qualitative work, this study was subject to social desirability bias. More specifically, participants in multidisciplinary focus groups might refrain from expressing something that could portray them in an unfavorable light. Fourthly, the experience gained during the study allowed us to make the questions increasingly less subjective. Whenever a new focus group was transcribed, errors in the way it was conducted were noted and subsequently corrected. Fifthly, in order to minimize confirmation bias, the investigators’ preconceived ideas were surveyed prior to the study. Since the investigators came from different professions, it was possible to fully cross-check the comments made in the focus groups. Lastly, to reduce interpretation bias, two focus groups were annotated independently by two investigators. For the three other focus groups, the passages that appeared to be most open to interpretation were annotated independently by two or even three investigators.

To the best of our knowledge, the present study is the first to have analyzed focus groups comprising all the professionals involved in the transmission and implementation of medication reconciliations. This enabled us to address various points of view and to identify common themes and differences expressed by the participants.

## Data Availability

The datasets generated during the current study are not publicly available due to containing information that could compromise the privacy of research participants but are available from the corresponding author (celine.rahuel@etu.univ-tours.fr) on reasonable request.
